# “Normal” Hallucinations and Attention

**DOI:** 10.3389/fnins.2021.731600

**Published:** 2021-09-07

**Authors:** Talis Bachmann

**Affiliations:** School of Law and Institute of Psychology, University of Tartu, Tartu, Estonia

**Keywords:** hallucination, attention, expectation, perception, illusion

Usually, people perceive what is out there veridically^1^. While driving a car, we correctly see the road ahead and other cars moving (How could we otherwise survive). The better we attend, the better we perceive and safeguard adequate reactions. However, there are some contexts where veridical perception is disturbed and replaced by nonveridical conscious experience—the process by which the actually presented sensory signals that carry information about the objects becomes disturbed and turn to represent these objects nonveridically. In subjective perception, for example, some feature of an object can be nonveridically associated with some other feature of a different object, called the feature misbinding effect where illusory objects are experienced (Treisman and Schmidt, 1982; Wu et al., 2004; Zhang et al., 2014). Typically, overload of attention, extreme brevity of object in view, presence of noise, specific (biasing) context, sensory deprivation, idiosynchratic traits, and pathological state of the perceiver are the circumstances prone to cause perceptual illusions and other distortions of perceptual experience (Behrendt and Young, 2004; Collerton et al., 2005; Friston, 2005; Bell et al., 2006; Meppelink et al., 2010; Bachmann et al., 2011; Ward, 2013; Nour and Nour, 2015; O'Callaghan et al., 2017; Corlett et al., 2019; Horga and Abi-Dargham, 2019; Coren and Girgus, 2020). Circumstances leading to hallucinatory experiences belong to this set of contexts.

In the domain of nonveridical sensory experience, there is a principal difference between two types of nonveridicality. Borrowing from Macpherson and Batty (2016), we will stick to these two traditional definitions: (1) Illusion: you perceive an object but you *misperceive* one or more of its properties; (2) Hallucination: you have an experience *as of an object* and its properties *but there is no object*, and there are no properties, that you perceive in virtue of having that experience It must be noted, however, that the taxonomy of nonveridical experiences constituted by illusions and hallucinations is actually more fine-grained, leading possibly to more than 10 subtypes of nonveridicality. This is depending on the relative share of illusory distortion between object and its separate features and on how the hallucinatory surplus additions to experience apply to object and/or its separate features (Macpherson and Batty, 2016). For additional subtleties in specifying illusions, see also Todorović (2020). I will leave these levels of scrutiny aside for the time being.

Hallucinations belong to the generally acknowledged symptoms in the diagnosis of pathological neuropsychiatric conditions (despite that for the specialists, there is no clarity in distinguishing norm and pathology—Larøi, 2012; Rodríguez-Testal et al., 2021) and are often exclusively associated with illness. However, there are also contexts where subjects in a normal state of mind experience objects that are not actually present (Aru and Bachmann, 2017; Aru et al., 2018; Vetik et al., 2020). Are these aberrant positive percepts a result of mistakenly applied mechanisms of selective attention, therefore causing ignition of inappropriate perceptual representations; or are these “normal hallucinations” created by mechanisms responsible for contentful perception apart from attention mechanisms? Recently, for example, it has been proposed that hallucinations are related to mind wandering (Fazekas, 2021), which suggests that attention may be involved indeed as the wandering mind is by no means a well-focused mind.

One original paradigm allowing to explore this question was developed by Mack et al. (2016), although for different purposes—to test the phenomenal overflow notion with a modified Sperling paradigm typically used to study iconic memory. They manipulated attention in a dual-task experimental design. According to the main task, participants had to report the features of the four circles in the four corners of the display. On a small proportion of trials, the centrally located letters had to be reported (which was a second type of task). Participants had to attend to one of the tasks, indicated by a postcue (a high or low pitch tone). Unexpectedly and without informing the participants about this, on the 101st trial, there were no central small letters on the screen and the postcue (the tone) directed the participants to the circle task. Furthermore, after such additional postcue, they were nevertheless asked to enter the letters (which had not been there actually). Importantly, majority of the participants nevertheless entered letters on this (actually, a letter-wise empty) trial (Mack et al., 2016, interpreted the results as indicative of absence of phenomenal overflow—subjective experience of perceptual content that could not be reported—as the participants must have experienced the absence of letters if their iconic memory was veridically present). Aru and Bachmann (2017) replicated this design and asked participants to rate the subjective clarity of the central letters when they were actually absent in the display. Results showed prevalence of illusory (“normally hallucinated”) experience of the actually absent stimuli in a substantial part of the participants. While those who did not experience hallucinations at all rated visibility at zero level, hallucinating participants gave visual subjective clarity ratings for these “ghost” letters at low or intermediate magnitude levels. Presumably, the repetitive presentation of central letters in earlier trials either did build up perceptual expectation and top–down processing or Pavlovian-type associative learning mechanisms caused nonveridical perception (on these mechanisms having an effect also at the early cortical level—Kok et al., 2013; Summerfield and de Lange, 2014; Powers et al., 2016; Corlett et al., 2019). Importantly, this effect happened when attention was directed to the different tasks with task-relevant stimuli located in different (more peripheral) areas of the display (Analogous results where audiovisual associations that caused auditory hallucinations were reported by Powers et al., 2017).

In a follow-up study, Aru et al. (2018) replicated these results and extended the normal hallucinations effect to a slightly different experimental design (with more than 90% of participants experiencing actually absent objects at least once). In the first experiment, a centrally precued (80% valid, 20% invalid) face was briefly presented either to the right or left from fixation; an outline of a faint square surrounded the face spatially. The participant either had to indicate the gender of the face or rate the subjective visibility of the square on a 4-point scale. In the first part of the experiment, i.e., the single task conditions, tasks are trained independently. In the main part (dual-task condition), the participant had to be prepared for all tasks and the face task was prompted on 90% of trials, which draw attention away from the square stimulus. In the critical trials (six times per participant), square was not depicted around the face, but the participants were nevertheless postinstructed to rate the visibility of the absent square. Only one out of 14 participants did not experience hallucination (rating 1); six participants hallucinated on more than three occasions; and nine participants at least once used the rating 3 (“almost clear experience”) for the absent stimulus. Endogenous attentional precueing had a significant facilitatory effect on face gender discrimination task, but the precueing effect on whether participants hallucinated or not was not found. Conscious experience of some absent object could not be predicted based on whether spatial attention was directed at a closely neighboring, task-relevant object.

In Experiment 2a (Aru et al., 2018), upright or inverted faces without surrounding frames were shown on the sides of the display and a small Landolt stimulus was presented in the middle. The participant either had to indicate whether the faces have a similar orientation or rate the visibility of the Landolt (central fixation was necessary across all trials). Again, in the main experimental phase (dual-task condition) in the dual-task condition, the participants had to perform the face task on 90% of trials; in the critical trials, there was no Landolt, but visibility rating for this physically absent stimulus was requested. In the critical trials, among the 17 participants, only one never hallucinated; six participants reported illusory perception every time; 12 participants at least once used the rating—“almost clear impression …” and six participants reported to have “a clear impression…” of the missing stimulus; on a 67% of critical trials some experience of the stimulus was reported. A significant difference between visibility ratings when the square was absent (2.25) compared with when it was present (2.98) showed a less-distinct perception for the hallucinated stimulus compared with the actually presented stimulus.

The results introduced above acknowledge certain regularities concerning the relation between attention and normal hallucinations. First, people tend to hallucinate in dual-task conditions when task attention is directed to an object different from another, task-irrelevant object that is conditioned to be associated with it. Therefore, illusory conscious experience of an actually absent object is not very likely to be ignited when attention is directed to expect it (This adds to some earlier experimental results showing subduing effects of selective attention on the duration of visual aftereffects—Bachmann and Murd, 2010; Murd and Bachmann, 2011. However, in the case of normal hallucinations, mutual autonomy of attention and consciousness mechanisms is indicated by the null effect of attention on hallucinations). Second, these hallucinated experiences of mentally normal people are not associated with some specific experimental design but can be found by different experimental designs such as iconic memory postcueing, spatial-attentional precueing, and spatially divided attention without precueing (The last mentioned variety of design allows disentangling normal hallucinations from the eye-movement mechanisms). Third, as for the measurement of hallucinatory experiences, direct subjective clarity evaluation methods were used; we remain agnostic with regard to this important open question: whether reporting normal hallucinations may have been produced as biased responding due to high base rate of the actually present stimuli in early trials. For instance, even with eyes closed, some participant could respond with rating “vague impression” because (s)he expects the stimulus to be presented. Similarly, because (s)he may use very lax criterion even with eyes open and decides to evaluate sensory noise as signal, thus producing a false alarm. Our intuition and also our first-person phenomenal experience in our first pilot experiments when we set to test the Mack et al. (2016) iconic memory paradigm dispose us to reject the response bias interpretation. Moreover, as explained in Aru et al. (2018), standard signal detection theory-based analysis cannot be used for our paradigms because it is ambiguous whether to regard hallucination as noise or as a signal when we want to investigate phenomenal, first-person conscious experience. In one way or another, hallucination is a reality, even though a phenomenal one.

In our subsequent studies, normal hallucinations were documented regularly and shown to be independent of some personality characteristics such as interrogative suggestibility and higher-level metacognitive self-evaluation (Taal and Bachmann, 2020; Vetik et al., 2020, where out of 35 participants, 33 hallucinated on at least one trial). One finding pointing at a possible association of normal hallucinations with individual differences pertains to one of the earlier experiments (Aru et al., 2018). Namely, there was a significant negative correlation between the Autism Spectrum Quotient score and frequency of normal hallucination. Why this effect was not found in other experiments where ASQ score was used as an independent variable is not clear. Possible “suspects” here are underpowered experiments and/or differences in the experimental designs. On the other hand, the issue of relation between autistic traits and propensity to experience illusions and hallucinations is far from being fully understood anyway (Pellicano and Burr, 2012; Lawson et al., 2014; Van de Cruys et al., 2014; Palmer et al., 2017; Utzerath et al., 2019).

To hypothesize on the likely level of processing where the type of perceptual hallucination described in this paper is produced, I will turn first to findings by Taal and Bachmann (2020) and Vetik et al. (2020). As verbally presented biasing suggestions in the interrogative suggestibility measurement did not correlate with propensity to hallucinate (Taal and Bachmann, 2020), normal hallucinations are probably not caused by verbal instructions describing the letter task; this is moreover so because this instruction was not repeated many times. Likely, lack of involvement of higher cognitive levels of metacognition in predisposing people to normal hallucinations is consistent also with absence of correlation between self-rating of one's ability to self-evaluate and propensity to hallucinate (Vetik et al., 2020). However, as Vetik et al. (2020) found a negative correlation between hallucination proneness and self-confidence in performance on the face recognition task, this hints at the involvement of some perceptual level of processing and not metacognitive decision-making level.

Finally, in order to characterize the possible content type of normal hallucinations, we turn again to the taxonomy suggested by Macpherson and Batty (2016) with an excerpt on hallucinations:

(ix) *hallucination of an object and veridical perception of the property of some object, experienced as a property of the hallucinated object*—(1) in case of the Mack task location and general outline of the letter matrices, but not clear letter identities experienced; (2) in case of Aru et al. (2018) tasks, the presence of the square object with clarity is less than when experienced with objectively real presentation (but we cannot know how distinct the experience was in terms of the criteria contents used by the participants for ratings);

(x) *hallucination of an object and illusory perception of the property of some object, experienced as a property of the hallucinated object*—(1) not very likely because in this case, participants could have noticed something unusual or odd; (2) possible nonveridical experience of some letter not actually presented among letters in the experiment;

(xi) *hallucination of an object and hallucination of a property, experienced as a property of the hallucinated object*—overwhelming. It is the agenda of future experiments to disentangle the subjective content of normal hallucinations as dependent on specific perceptual and personality-trait contexts.

## Author Contributions

The author confirms being the sole contributor of this work and has approved it for publication.

## Conflict of Interest

The author declares that the research was conducted in the absence of any commercial or financial relationships that could be construed as a potential conflict of interest.

## Publisher's Note

All claims expressed in this article are solely those of the authors and do not necessarily represent those of their affiliated organizations, or those of the publisher, the editors and the reviewers. Any product that may be evaluated in this article, or claim that may be made by its manufacturer, is not guaranteed or endorsed by the publisher.

## Footnotes

^1^“‘Veridicality' is used in this paper with reference to objective *external* veridicality, i.e., reality of the actual environmental source of these experiences. This is usually established by verifiable facts from the third-person observations that there is a one-to-one correspondence between the external object and its categorical reference by the subject (In terms of subjective reality from the first-person point of view, all experiences are veridical, whether truthful or not from the objective-veridicality point of view).”
